# Sex Differences in Social Attention in Infants at Risk for Autism

**DOI:** 10.1007/s10803-018-3799-z

**Published:** 2018-11-22

**Authors:** Johan Lundin Kleberg, Pär Nyström, Sven Bölte, Terje Falck-Ytter

**Affiliations:** 1 Department of Psychology, Uppsala Child and Baby Lab, Uppsala University, Box 1225, 751 42 Uppsala, Sweden; 2 Department of Clinical Neuroscience, Karolinska Institutet, Stockholm, Sweden; 3 Department of Women’s and Children’s Health, Center of Neurodevelopmental Disorders (KIND), Karolinska Institutet, Stockholm, Sweden; 4 Child and Adolescent Psychiatry, Center for Psychiatry Research, Stockholm County Council, Stockholm, Sweden; 5 The Swedish Collegium for Advanced Study (SCAS), Uppsala, Sweden

**Keywords:** Autism Spectrum Disorder (ASD), Eye tracking, High-risk infants, Emotion, Broader autism phenotype, Face processing

## Abstract

We studied visual attention to emotional faces in 10-month-old infant siblings of children with ASD (ASD-sibs; *N* = 70) and a siblings of typically developing children (*N* = 29) using static stimuli. Contrary to our predictions, we found no evidence for atypical gaze behavior in ASD-sibs when boys and girls were analyzed together. However, a sex difference was found in ASD-sibs' visual attention to the mouth. Male ASD-sibs looked more at the mouth across emotions compared to male controls and female ASD-sibs. In contrast, female ASD-sibs looked *less* at the mouth compared to female controls. These findings suggest that some aspects of early emerging atypical social attention in ASD-sibs may be sex specific.

**Electronic supplementary material:**

The online version of this article (10.1007/s10803-018-3799-z) contains supplementary material, which is available to authorized users.

Siblings of children with autism spectrum disorder (ASD) are a population with highly elevated prevalence of ASD, as well as other forms of neurodevelopmental and psychiatric challenges. Longitudinal studies following this population (hereafter ASD-sibs) from infancy to early childhood have shown that around 25% of male infants and 10% of female infants in this group are later diagnosed with ASD as compared to around 1–2% in the general population (Idring et al. 2015; Messinger et al. 2015; Ozonoff et al. 2011). ASD-sibs who do not fulfill the full criteria for an ASD diagnosis often have elevated levels of subclinical ASD symptoms (Messinger et al. 2013), or other clinical conditions, including language disorders, ADHD, externalizing and internalizing disorders. (Jones et al. 2014; Messinger et al. 2013; Ozonoff et al. 2011). Therefore, comparisons between ASD-sibs and infant siblings of children without familiar risk for ASD can provide new leads on potential early differences related to later neurodevelopmental and psychiatric problems in general, and ASD in particular.

In the present study, we focused on visual attention to faces in ASD-sibs and a control group of infants without familiar risk for ASD. There are at least two important reasons to study this topic. First, reduced or atypical attention to social information such as faces and biological motion is commonly seen in toddlers and young children with ASD (Chawarska and Shic 2009; de Wit et al. 2008; Falck-Ytter et al. 2013; Guillon et al. 2014; Kleberg et al. 2017; Moriuchi et al. 2017), although existing research points to a considerable variation across experimental tasks (e.g. Guillon et al. 2014; Falck-Ytter and von Hofsten 2011). Since ASD-sibs are at high risk for ASD and autistic symptoms, early atypical attention to faces could represent an early sign of ASD symptomatology. Secondly, visual attention to faces is likely to be highly important in infant development beyond core ASD symptomatology. Previous studies have documented that typically developing infants are highly attentive to faces (e.g. Bakker et al. 2011; Gredebäck et al. 2012; Oakes and Ellis 2013), which in turn is linked to the development of social cognition, as well as to language acquisition (e.g. Tenenbaum et al. 2013; Gredebäck et al. 2012), and development of the social brain (e.g. Johnson et al. 2015). Early atypical attention to faces could therefore have cascading consequences that ultimately lead to the development of behavioral difficulties. A better understanding of visual attention to faces in ASD-sibs can therefore contribute to an understanding of the early development of ASD-sibs in multiple areas. In the following sections, we briefly review the literature about visual attention to faces in infants without elevated risk for ASD, before turning to the literature about visual attention to faces in ASD-sibs.

## Visual Attention to Faces in Infancy

Face scanning undergoes a rapid development during the first year of life in typically developing infants. At 4 months, infants direct their visual attention mainly to the eyes and relatively little to the mouth. Although eyes and mouth region doubtlessly continue to be important sources of social information from infancy and throughout development, a relative shift towards the mouth and a gradual decrease in attention to the eyes is seen during the second half of the first year, that reaches its peak levels between 8 and 12 months (Lewkowicz et al. 2012; Oakes and Ellis 2013; Tenenbaum et al. 2013). This increase in looking time at the mouth has been related to language acquisition (Lewkowicz et al. 2012).

During the second half of the first year, infants also develop an increasing ability to differentiate between facial emotions. Whereas infants are sensitive to facial expressions of happiness already during the first weeks of life (Field et al. 1982; Grossmann et al. 2007), fearful faces are reliably detected from around 7 months of age (see Leppänen and Nelson 2012 for a review). Eye tracking studies have shown that infants from 7 months of age look longer at fearful faces as compared to faces displaying other emotions (Peltola et al. 2008), and also distribute their visual attention more broadly between areas within faces with a fearful expression as compared to other expressions (Hunnius et al. 2011; Gredebäck et al. 2012). As a consequence, infants may look *less* at the eyes of fearful faces, as compared to happy or neutral (Hunnius et al. 2011). The fact that looking time at the eyes is decreased for fearful faces may seem counterintuitive in light of other studies showing that the eyes are typically the most diagnostic region for identifying fear (Adolphs 2008), but may represent a ‘vigilant’ form of attention driven by the potential presence of a threat (Hunnius et al. 2011; Gredebäck et al. 2012). To sum up, infants are attentive to faces during the first year, and their visual attention becomes increasingly sensitive to the emotional valence of the faces.

Sex differences in some aspects of face processing have been found in typical development. For example, Pascalis et al. (1998) reported earlier maturation of face processing in 3–6 month old male infants, and Rennels and Cummings (2013) reported differences in visual scanning strategies of female and male infants at 3–6 and 9–10 months. In this study, male infants were more likely than female infants to shift their gaze between external and internal features of the face, whereas female infants made more gaze shifts within the internal regions of the face. Sex differences in visual scanning could relate to differences in cognitive processing. For example, female infants have often been found to perform better in face recognition tasks (McClure 2000).

## Visual Attention to Faces in ASD-Sibs

A number of previous studies have examined visual attention to faces in ASD-sibs. Of these, studies using static images of smiling or neutral faces as stimuli found highly similar visual scanning of core regions in ASD-sibs and controls during the first year of life (Dundas et al. 2012; Key and Stone 2012; Young et al. 2009). The aforementioned studies have compared ASD-sibs to control groups without familiar risk for ASD. Further, longitudinal studies have examined whether visual attention to faces in ASD-sibs predicts a diagnosis of ASD: a recent study reported that six month old ASD-sibs who were later diagnosed with ASD did not differ from a matched control group in overall looking time at images of mothers’ and strangers faces. However, ASD-sibs who were *not* later diagnosed with ASD looked less at the stimuli than both ASD-sibs with a later diagnosis and controls (Wagner et al. 2016). In contrast, a small number of eye tracking studies using dynamic videos as stimuli have found atypical face scanning in infants later diagnosed with ASD (Chawarska et al. 2013; Jones and Klin 2013; Shic et al. 2014). To our knowledge, only one study has examined the effect of facial emotion on visual attention in ASD-sibs. Wagner et al. (2016) examined looking time to happy, fearful and neutral faces in a group of 9 month old ASD-sibs who did not fulfill the criteria for an ASD diagnosis at a subsequent 36 months visit and a typically developing control group. Both groups looked more at the eyes of fearful faces and more at the mouth of happy faces. In addition, the ASD-sibs who did not develop ASD had larger pupil dilation (an index of autonomic nervous system arousal) when viewing faces than controls, regardless of emotional expression (Wagner et al. 2016).

As noted previously, typically developing infants increase their looking time at the mouth of faces during the second half of the first year. This change is believed to be related to verbal development. An interesting question is therefore whether the same relation between visual attention to the mouth area of faces and concurrent or later language ability is seen in ASD-sibs. In support of this hypothesis, two studies have reported that attention to the mouth in ASD-sibs at nine (Elsabbagh et al. 2014) and six (Young et al. 2009) months predicts later expressive language skills at 24–36 months in both ASD-sibs and controls. Another study found that more gaze at the eyes at 6 months predicted worse expressive language at 24 months ASD-sibs but not in controls (Wagner et al. 2016). Together, these studies suggest that individual differences in language acquisition may be related to face scanning in both ASD-sibs and controls, but that the relationships may be different in the two populations.

### Sex Differences in ASD-Sibs

Male ASD-sibs are at a two- to threefold risk of ASD as compared to female ASD-sibs (e.g. Messinger et al. 2016; Ozonoff et al. 2011). This has led to an interest in sex differences in early social attention in this group. One aim of this line of research is to identify potential compensatory mechanisms or protective factors against ASD in female infants. For example, Chawarska et al (2016) reported that female ASD-sibs (regardless of subsequent diagnostic outcome) looked longer at the face of a speaking actress both compared to male ASD-sibs and control infants. Increased attention to faces was associated with better socio-communicative skills at 24 months in both sexes, as measured with the Autism Diagnostic Observation Schedule (ADOS). This suggests that visual attention to the eyes may be related to protective or compensatory processes. It is also possible that partly *different* mechanisms may lead to ASD in male and female infants. A recent study by Bedford et al. (2016) examined the longitudinal predictive relationships between three previously identified behavioral markers of ASD at 14 months and autistic symptoms at 36 months broken down by sex. The three markers represented non-social attention (visual disengagement), social attention (gaze following) and a composite symptom measure (autism observation scale for infants; AOSI, Bryson et al. 2008). Previous studies have reported that these three tasks predict an ASD diagnosis in ASD-sibs, but Bedford et al. (2016) reported that these relations were only found in males. Taken together, these studies suggest that sex differences are important to examine in studies of ASD-sibs. However, it should be noted that, since the base rate of ASD symptoms is higher in males, studies of sex differences in young infants with ASD or ASD-sibs typically have lower power to detect atypicalities in females.

### Aims and Hypotheses

The present study was designed to compare visual attention to emotional faces in 10 month old ASD-sibs to a control group without family risk for ASD. In line with previous studies in infants, we expected fearful faces to elicit lower relative looking time at the eyes than happy faces. We hypothesized that this effect of emotion would be smaller in ASD-sibs than in controls. In light of recent reports of sex differences in attention and developmental pathways in ASD-sibs (e.g. Chawarska et al. 2016), sex was added as a factor in all analyses, but we did not have an a priori hypothesis related to this factor. Similarly, previous studies indicate that there may be differences between typical infants and ASD-sibs in terms of looking time to eyes and mouth (e.g. Jones and Klin 2013; Chawarska et al. 2013), but here, again, we did not specify the direction of these results as previous data are rather mixed. Finally, we analyzed linear relationships between looking time at the eyes and mouth, and the expressive and receptive language subscales of the Mullen Scales of Early Learning (MSEL; Mullen 1995). We consider these analyses exploratory.

## Methods

### Participants

Data from 99 infants (70 ASD-sibs) were included in the analysis. Data were collected as part of an ongoing longitudinal study following infants from the first year of life (The Early Autism Sweden (EASE) study; http://www.smasyskon.se). All infants in the ASD-sibs group had one or more full siblings with a community diagnosis of ASD. The diagnosis of the older sibling was confirmed through consultation of medical records. The control group was recruited from a database of families who had expressed interest in developmental research. All infants in the control group had one or more sibling with typical development, and no family history of ASD up to second degree relative. All infants were born full term (> 36 weeks) and did not have any confirmed or suspected medical problems, including visual/auditory impairments. As can be seen in Table 1, the two groups did not differ in age at assessment, gender distribution, verbal, and non-verbal cognitive development, as measured with the MSEL. There were also no gender differences within either controls or ASD-sibs (lowest *p* = 0.19). In addition to the sample reported here (*N* = 99), six infants were initially tested but excluded from analysis, because they were half-siblings of an older child with ASD. One infant in the control group was excluded from the analysis because of a subsequent ASD diagnosis, and 14 infants (7 ASD-sibs) were seen but excluded because too little valid data was recorded (because of either equipment failure or calibration problems). One infant in the ASD-sibs group was considered an outlier in the eye-mouth index (see definition below) and was therefore excluded from further analysis (see "Data Reduction").

**Table 1:** Gender, age, and cognitive development

Measure	ASD-sibs (*N* = 70)	Control group (N = 29)	*p*
Gender (% Female)	52%	59%	.49^a^
Age (days); *M* (sd)	311.5 (11.4)	307.7 (14)	.17^b^
MSEL Early Learning Composite Raw Score; *M* (sd)	99.72 (13.51)	103.30 (11.40)	.20^b^
MSEL Visual Reception Raw Score; *M* (sd)	13.92 (2.17)	14.20 (1.19)	.545^b^
MSEL Fine Motor Raw Score; *M* (sd)	13.63 (1.73)	14.12 (1.20)	.211^b^
MSEL Expressive Language Raw Score; *M* (sd)	10.04 (1.97)	10.33 (2.23)	.244^b^
MSEL Receptive Language Raw Score; *M* (sd)	10.80 (2.10)	10.53 (2.08)	.453^b^

*MSEL* Mullen Scales of Early Learning

Numbers represent raw scores

^a^*Χ*^2^-test (two-tailed)

^b^t-test (two-tailed)

### Ethics Approval and Consent to Participate

Parents provided written informed consent, and the study was approved by the Regional Ethical Board in Stockholm. The study was conducted in accordance with the standards specified in the 1964 Declaration of Helsinki.

### Data Collection and Analysis

Infants watched the stimuli seated in the lap of a parent. Stimuli were presented on a computer monitor placed at approximately 60 cm distance. The experimental stimuli were presented in random order interleaved with stimuli from other experiments (including inverted faces) not analyzed here. Gaze data were recorded using Tobii corneal reflection eye trackers (Tobii Technology, Danderyd, Sweden). A change in equipment took place during the period of data collection as follows: data from 48 infants (34 ASD-sibs) were recorded at a sample rate of 50 Hz with a Tobii 1750 (Tobii Inc, Danderyd, Sweden; Screen resolution: 1280 × 1024 pixel; Screen size: 17″), and data from 17 infants (11 ASD-sibs) were recorded at 120 Hz, and from 34 infants (25 ASD-sibs) at 300 Hz with a Tobii TX300 system (Tobii Inc, Danderyd, Sweden; Screen resolution: 1600 × 1200 pixels; Screen size: 23″). Stimuli were presented in the same size (342 × 274 mm) on both eye trackers.

No difference was found in the proportion of ASD-sibs and controls, *χ*^*2*^ (1) = 0.004, *p* = 0.948, or the proportion of boys and girls, χ^2^ (1) = 0.482, *p* = 0.488, tested with the two eye tracking systems. The proportion of rejected samples was slightly higher in the older T1750 eye tracker, but the difference was not significant, *t* (96) = 1.68, *p* = 0.089. To control for potential equipment differences, all analyses were calculated with eye tracker (T1750, TX300) included as a fixed effect. No significant main or interaction effect involving eye tracker was found (lowest *p* = 0.58). We therefore pooled the data, and eye tracker was excluded as covariate in the final model. A cognitive assessment with the MSEL was performed during the same visit as the eye tracking experiment.

### Stimuli

Stimuli consisted of static images of adult faces from the Karolinska Directed Emotional Faces library (Lundqvist et al. 1998) of female and male models displaying either a fearful or a happy expression. During the experiment, infants saw four fearful and four happy faces, and each image had a presentation duration of 5 s. For each infant, the images presented were randomly selected from a larger set of 16 stimulus pictures. All trials were preceded by a moving animation in order to attract the infant’s attention to the center of the screen, and presented for 5 s. Due to a technical error, 24 infants (20 ASD-sibs) saw five instead of four presentations. In these cases, the fifth trial was removed from further analysis to ensure that the maximum number of analyzed trials was equal between participants. The proportion of male stimulus faces was 53% in the ASD-sibs group and 51% in controls.

### Data Reduction

Raw gaze coordinates were analyzed using custom scripts written in MATLAB (Mathworks Inc., CA, USA). Average values for the right and left eye were used in the analysis. Standard fixation parsing algorithms may not be reliable in infants (e.g. Wass et al. 2013). Therefore, we analyzed accumulated looking time based on the raw data. To compensate for data loss due to movement artefacts and blinks, we interpolated linearly over gaps in the data shorter than 150 ms. In order to reduce noise (i.e. rapid changes in gaze position that are likely technical artefacts), data were filtered using a moving median filter with a window corresponding to 80 ms.

**Fig. 1:**
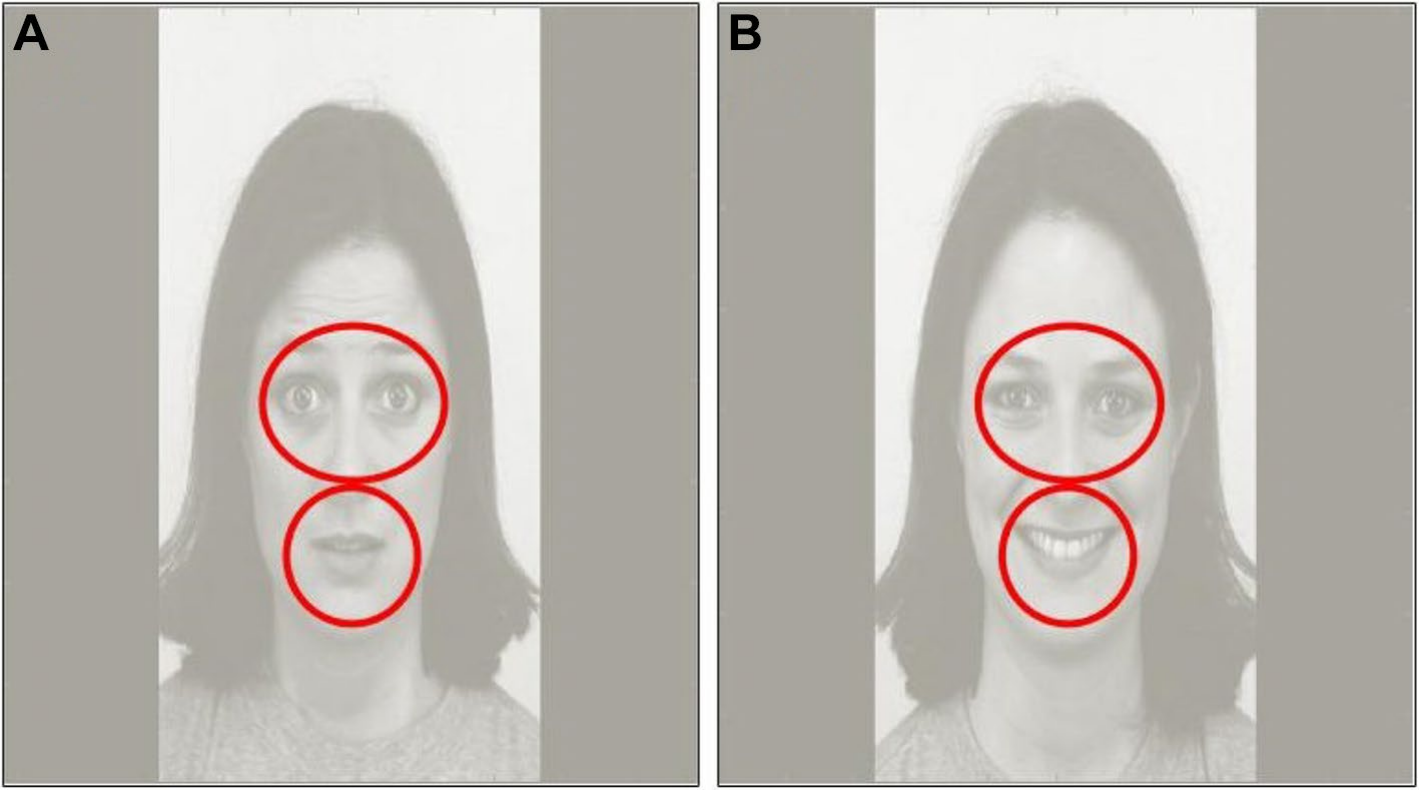
Areas of interest (AOIs) shown on a fearful (**a**) and happy (**b**) stimulus image

Trials with less than 750 ms valid looking time (15% of the trial) were discarded. With these criteria, 494 trials from 71 ASD-sibs (34 male; average proportion of valid trials per participant: 91%) and 204 trials from 29 controls were included 12 male; average proportion of valid trials per participant: 92%. We defined areas of interest (AOIs) covering the (1) whole screen; (2) the eyes; and (3) the mouth (see Fig. 1).

### Dependent Variables

The dependent variables were (1) total looking time at the screen in milliseconds; (2) looking time at the eyes (relative to total looking time at the screen), (3) looking time at the mouth (relative to total looking time at the screen), and (4) the eye-mouth index (henceforth, EMI), defined as looking time at the eyes divided by the summed looking time at the eyes and mouth. Higher EMI values therefore indicate more looking time at the eyes, relative to the mouth. The EMI is an index of the relative distribution of gaze between the eyes and mouth, and can therefore contribute additional information about gaze behavior during face perception. The EMI has been used in many previous studies of face scanning in ASD and ASD-sibs, and gives a composite measure of the relative distribution of gaze within the face (e.g. Falck-Ytter 2008; Young et al. 2009; Merin et al. 2007).

### Statistical Analysis

All statistical analyses were performed in MATLAB (version 2016b, Mathworks, Inc.). Prior to analyses, all variables were inspected for outliers, both in terms of single responses and average responses for each participant. One female participant in the ASD-sibs group was excluded from analyses of the eyes, mouth, and EMI variables because of an average value deviating more than three standard deviations from the mean of the full sample as well as in the group of female ASD-sibs. The EMI and mouth variables were negatively skewed, and were therefore arcsine transformed. When the analyses were performed on the untransformed data, all significant effects remained unchanged. Data were analyzed using linear mixed effects (LME) models using the ‘fitlme’ function with random intercepts for subject. Emotion (happy, fearful), group (ASD-sibs, controls), and gender (male, female), were fixed factors (predictors). LME models are useful for analyzing data with inter-individual variability and uneven number of trials between participants (Baayen et al. 2008) and have been used in previous infant eye tracking studies (Chawarska et al. 2016).

Since previous literature has suggested that language development is strongly related to face scanning at 10 months and infants varied widely in language level, we added the expressive and receptive language subscales of the MSEL as predictors in all analyses. However, all the reported significant results remained when these covariates were removed from the models. We tested significant effects by comparing models with and without the fixed effects using likelihood ratio tests (LRT) computed with the ‘compare’ function in MATLAB (see Baayen et al. 2008). In preliminary analyses, we also added trial number, model gender (male, female), and MSEL Fine Motor and Visual Reception scores as predictors. No significant main or interaction effects involving these measures were found (lowest *p* = 0.09), and these covariates were therefore dropped from the main analysis. Residual plots indicated that residuals were approximately normally distributed in all analyses.

## Results

### Preliminary Analysis

Looking time at the whole screen decreased in later trials, *χ*^2^ (1) = 27.35, *p* ≤ 0.001, *b* = − 201.36, *SE* = 37.77, but there were no interactions between trial and group, *χ*^2^ (1) = 0.18, *p* = 0.672, trial and emotion, *χ*^2^ (1) = 1.57, *p* = 0.210, or trial and sex, *χ*^2^ (1) = 0.15, *p* = 0.694. There were also no three- or four-way interactions between trial order, and looking time at the screen (all *p* > 0.20). Looking time at the eyes decreased in later trials, *χ*^2^ (1) = 7.24, *p* = 0.007, *b* = − 0.02, *SE* = 0.01, but there was no interaction between trial and group, *χ*^2^ (1) = 0.37, *p* = 0.542, trial and sex, *χ*^2^ (1) = 2.12, *p* = 0.146, or trial and emotion, *χ*^2^ (1) = 1.62, *p* = 0.204. No relation was found between trial order and looking time to the mouth or EMI (*p* > 0.07).

### Screen

Looking time at the screen was not related to emotion, *χ*^2^ (1) = 0.03, *p* = 0.861, *b* = − 14.36, *SE* = 81.91, group, *χ*^2^ (1) = 1.07, *p* = 0.300, *b* = 180.20, *SE* = 173.49, or sex, *χ*^2^ (1) = 0.13, *p* = 0.718, *b* = 56.47, *SE* = 156.47. No significant interactions were found between group and emotion, *χ*^2^ (1) = 0.01, *p* = 0.929, *b* = 16.15, *SE* = 180.17, group and sex, *χ*^2^ (1) = 0.13, *p* = 0.722, sex and emotion, *χ*^2^ (1) = 0.11, *p* = 0.741, or group, sex, and emotion, *χ*^2^ (1) = 1.58, *p* = 0.209.

### Eyes

**Fig. 2:**
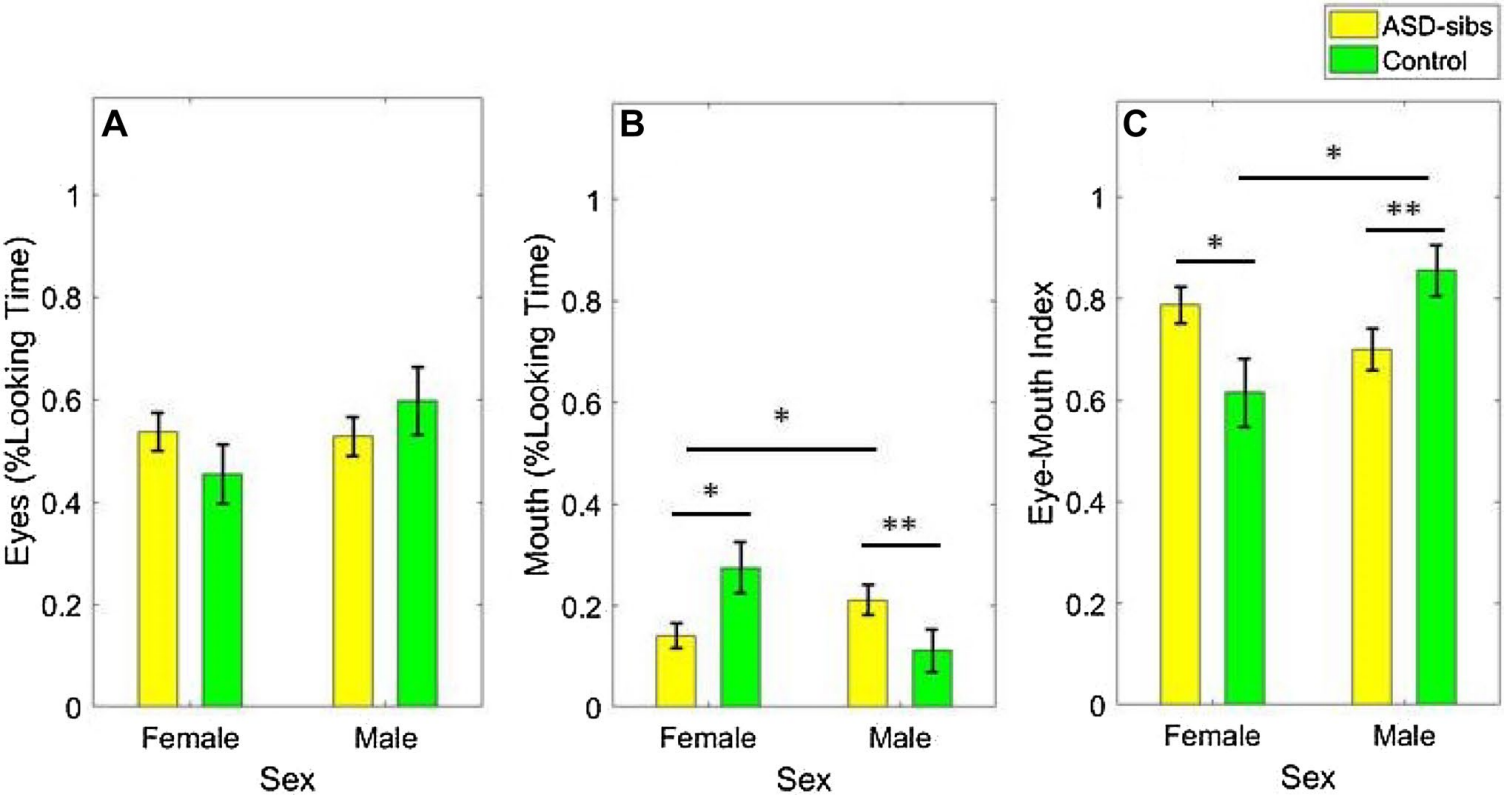
Average proportion of looking time to the eyes (**a**), mouth (**b**), and Mean Eye-Mouth Index (EMI); **c** in ASD-sibs and controls as a function of sex. Error bars represent 95% confidence intervals of the mean. *p < 0.05, **p < 0.01

For looking time at the eyes, we found no significant main effects of group, *χ*^2^ (1) = 0.01, *p* = 0.909, *b* = − 0.01, *SE* = 0.05, emotion, *χ*^2^ (1) = 0.47, *p* = 0.492, *b* = 0.01, *SE* = 0.02, or sex, *χ*^2^(1) = 0.22; *p* = 0.641; *b* = 0.02; *SE* = 0.04. There were also no significant interaction effects between group and emotion, *χ*^2^ (1) = 0.05, *p* = 0.816, sex and group, *χ*^2^ (1) = 1.82, *p* = 0.177, or sex and emotion, *χ*^2^ (1) = 1.05, *p* = 0.306. These data are shown in Fig. 2.

### Mouth

There were no significant main effects of group, *χ*^2^ (1) = 0.11, *p* = 0.736, *b* = 0.04, *SE* = 0.1*2*, or sex, *χ*^2^ (1) = 0.21, *p* = 0.650, *b* = 0.05, *SE* = 0.11. However, we found a significant main effect of emotion, *χ*^2^ (1) = 8.64, *p* = 0.003, *b* = − 0.11, *SE* = 0.04, driven by lower proportion of looking time to the mouth of happy faces. Contrary to our predictions, we found no interaction effect between group and emotion, *χ*^2^ (1) = 2.65, *p* = 0.104. Since we had an a priori hypothesis about these results, we ran the analysis in the two groups separately. When the analysis was broken down by group, a significant effect reflecting lower proportion of looking time to the mouth of happy faces was found in the control group, *χ*^2^ (1) = 8.18, *p* = 0.004, *b* = − 0.20, *SE* = 0.07. This effect was not significant, but in the same direction, in ASD-sibs, *χ*^2^ (1) = 2.62, *p* = 0.106, *b* = − 0.07, *SE* = 0.04.

There was a significant interaction effect between sex and group, *χ*^2^ (1) = 8.44, *p* = 0.004. No significant interaction effect was found between sex and emotion, *χ*^2^ (1) = 0.12, *p* = 0.733, or emotion, sex, and group, *χ*^2^ (1) = 0.18, *p* = 0.671. Follow-up comparisons showed that male ASD-sibs looked more at the mouth than male controls, *χ*^2^ (1) = 7.01, *p* = 0.008, *b* = − 0.45, *SE* = 0.16, but that female ASD-sibs looked less at the mouth than female controls, *χ*^2^ (1) = 5.40, *p* = 0.020, *b* = 0.36, *SE* = 0.15. Within the ASD-sibs group, females looked less at the mouth than males, *χ*^2^ (1) = 4.01, *p* = 0.045, *b* = 0.24, *SE* = 0.12. In controls, a trend towards longer looking time at the mouth in females was found, *χ*^2^ (1) = 3.46, *p* = 0.063, *b* = − 0.39, *SE* = 0.20. These data are shown in Fig. 2.

### Eye-Mouth Index

Analyses of the EMI yielded highly similar results as the analyses of looking time at the mouth. No main effects of group, *χ*^2^ (1) = 0.02, *p* = 0.885, *b* = − 0.02, *SE* = 0.16, or sex, *χ*^2^ (1) = 0.14, *p* = 0.704, *b* = − 0.06, *SE* = 0.15 were found. The EMI was higher (i.e. longer looking time at the eyes relative to the mouth) when infants watched happy as compared to fearful faces, *χ*^2^ (1) = 4.77, *p* = 0.029, *b* = 0.10, *SE* = 0.05. We found no interaction effect between emotion and group, *χ*^2^ (1) = 0.91, *p* = 0.340. When the analysis was broken down by group, a significant effect of emotion reflecting higher EMI values for happy faces was found in the control group, *χ*^2^ (1) = 3.89, *p* = 0.048, *b* = 0.17, *SE* = 0.08, but not in ASD-sibs, *χ*^2^ (1) = 1.74, *p* = 0.187, *b* = 0.07, *SE* = 0.05.

As in the analysis of the mouth AOI, we found a significant interaction effect between infant sex and group for the EMI index, *χ*^2^ (1) = 8.59, *p* = 0.003. No significant interactions were found between sex and emotion, *χ*^2^ (1) = 1.05, *p* = 0.306, or emotion, sex, and group, *χ*^2^ (1) = 0.14, *p* = 0.711. Follow-up comparisons showed that male ASD-sibs had lower EMI values than male controls, *χ*^2^ (1) = 7.35, *p* = 0.007, *b* = 0.64, *SE* = 0.23, but that female ASD-sibs had higher EMI values than female controls, *χ*^2^ (1) = 4.99, *p* = 0.026, *b* = − 0.47, *SE* = 0.20. Within the ASD-sibs group, a trend towards higher EMI values in females as compared to males was found, *χ*^2^ (1) = 3.63, *p* = 0.057, *b* = − 0.32, *SE* = 0.17, whereas lower EMI values were found in female than in male controls, *χ*^2^ (1) = 4.00, *p* = 0.045, *b* = 0.55, *SE* = 0.27. These data are shown in Fig. 2.

### Relations Between Face Scanning and Concurrent Language Development

In controls, looking time at the eyes was negatively related to expressive, *χ*^2^ (1) = 5.69, *p* = 0.017, *b* = − 0.05, *SE* = 0.02, but not receptive language, *χ*^2^ (1) = 1.29, *p* = 0.256, *b* = − 0.03, *SE* = 0.02. No significant relations were found between looking time at the mouth and expressive, *χ*^2^ (1) = 3.35, *p* = 0.067, *b* = 0.09, *SE* = 0.05, or receptive, *χ*^2^ (1) = 0.79, *p* = 0.373, *b* = 0.05, *SE* = 0.05, language. In ASD-sibs, looking time at the eyes was not significantly related to expressive language, *χ*^2^ (1) = 0.88, *p* = 0.348, *b* = − 0.01, *SE* = 0.01, or receptive language, *χ*^2^ (1) = 0.56, *p* = 0.455, *b* = − 0.01, *SE* = 0.01. We did also not find evidence for a relation between looking time at the mouth and either expressive, *χ*^2^ (1) = 0.01, *p* = 0.906, *b* = 0.00, *SE* = 0.03, or receptive language in ASD-sibs, *χ*^2^(1) = 0.01, *p* = 0.931, *b* = 0.00, *SE* = 0.03.

## Discussion

The aim of the present study was to examine visual attention to fearful and happy faces in infant siblings of children with ASD (ASD-sibs) and controls. Our hypothesis was that ASD-sibs would show reduced differentiation between emotional expressions in terms of visual attention. The data did not support the hypothesis, as we did not find the expected interaction between group and emotion. This finding points to an area of preserved face processing in ASD-sibs. Across groups, a higher proportion of looking time was directed at the mouth of fearful as compared to happy faces. However, it is notable that the difference between emotions was only marginally significant in ASD-sibs, despite a relatively large sample size, whereas a strong effect of emotion was found in the control group.

A rather strong sex difference was found in visual attention to the mouth, both in proportion of total looking time and relative to the eyes. Male ASD-sibs scanned the mouth region more than male controls and female ASD-sibs, both relative to the eyes and in proportion of total looking time at the screen. The reverse pattern was found in female ASD-sibs—i.e. reduced attention to the mouth compared to female controls. This means that in both sexes, atypical scanning of emotional faces was found, but in different directions compared to sex matched controls. Sex differences in social attention in ASD-sibs during infancy may reflect a compensatory mechanism in females, or a higher accumulated load of risk factors in males (Chawarska et al. 2016; Messinger et al. 2015; Robinson et al. 2013). Longitudinal studies are needed to determine how the present results relate to subsequent outcome.

We also found a sex difference within the control group, with female infants looking more at the mouth relative to the eyes than males. Sex differences in social attention have previously been reported in typically developing infants (e.g. Rennels and Cummings 2013; but see Bakker et al. 2011). It is possible that the observed sex difference in the control group may reflect differences in processing of the visual characteristics of faces (e.g. Rennels and Cummings 2013). Alternatively, it is possible that the observed sex difference in face scanning in the control group is sign of emerging sex differences in language development. Female infants tend to develop earlier in the domain of language (e.g. Messinger et al. 2015), and relatively more attention to the mouth is related to later expressive language skills (Lewkowicz et al. 2012). It should be noted, however, that no sex differences were found in the language measures in MSEL. Interestingly, we found that less extensive scanning of the eyes was related to concurrent expressive language in controls only, not in ASD-sibs, despite this sample being substantially larger.

Attention to both eyes and mouth is important for infant social development, but are likely to be related to different socio-cognitive processes. Whereas attention to the eyes provide opportunities to learn about other’s intentional states, and focus of attention (Batki et al. 2000; Senju and Csibra 2008), attention to the mouth at during the second half of the first year is related to language acquisition (Lewkowicz et al. 2012). A speculative interpretation of our results would therefore be that increased attention to eyes in female ASD-sibs reflect protective factor against social-cognitive impairments, but may also be predictive of worse language development. Consistent with this prediction, Carter et al. (2007) reported that female toddlers with ASD had lower language functioning than male toddlers with ASD (but see Reinhardt et al. 2015). This notion can be tested once follow up data from the current sample is available.

In conclusion, our results suggest that female and male infant siblings of children with autism attend differently to the eyes and mouth of emotional faces. These findings, particularly if corroborated by larger studies with later ASD outcome, could contribute to the understanding of the early development of infant siblings at risk for autism, and stress the importance of studying the development of ASD separately in females and males.

## Electronic supplementary material

Below is the link to the electronic supplementary material.

Supplementary material 1 (JPG 40 KB)
