# Supplementary figures and images for: Sex Differences in Social Attention in Infants at Risk for Autism
Source: J Autism Dev Disord. 2018 Nov 22;49(4):1342–51. doi: 10.1007/s10803-018-3799-z (PMC6450841; doi:10.1007/s10803-018-3799-z)

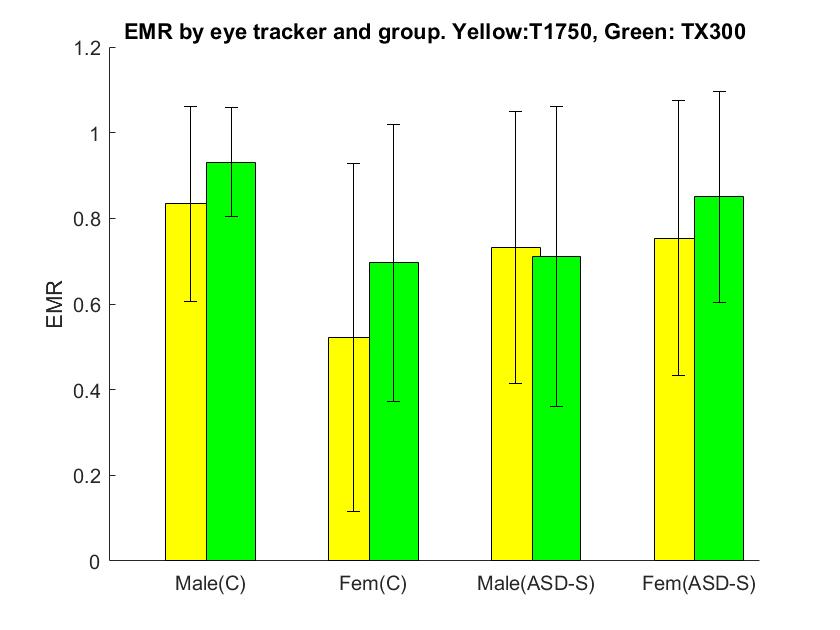

Supplement: Supplementary file 1 — Supplementary material 1 (JPG 40 KB) [file 10803_2018_3799_MOESM1_ESM.jpg]
